# 

**Published:** 1893-05

**Authors:** 


					﻿ESTABLISHED 1855.
SUBSCRIPTION, $2.00,,
ATLANTA
Medical and Surgical
JOURNAL.
wl. xX' L» Atlanta, Ga , May, i 893. No. 3>
LUTHER B. GRANDY, M. D.,	M. B. HUTCHINS, M. D.,
MANAGING EDITOR.	BUSINESS MANAGER.
				

